# Improving Linkage to HIV Care Through Mobile Phone Apps: Randomized Controlled Trial

**DOI:** 10.2196/mhealth.8376

**Published:** 2018-07-17

**Authors:** Willem Venter, Jesse Coleman, Vincent Lau Chan, Zara Shubber, Mothepane Phatsoane, Marelize Gorgens, Lynsey Stewart-Isherwood, Sergio Carmona, Nicole Fraser-Hurt

**Affiliations:** ^1^ Wits Reproductive Health and HIV Institute School of Clinical Medicine, Faculty of Health Sciences University of the Witwatersrand Johannesburg South Africa; ^2^ Department of Public Health Sciences Karolinska Institutet Stockholm Sweden; ^3^ World Bank Group Washington, DC United States; ^4^ National Health Laboratory Services Department of Molecular Medicine and Haematology, School of Pathology, Faculty of Health Sciences University of the Witwatersrand Johannesburg South Africa

**Keywords:** cell phones, app, Africa, linkage to care, HIV, patient information

## Abstract

**Background:**

In HIV treatment program, gaps in the “cascade of care” where patients are lost between diagnosis, laboratory evaluation, treatment initiation, and retention in HIV care, is a well-described challenge. Growing access to internet-enabled mobile phones has led to an interest in using the technology to improve patient engagement with health care.

**Objective:**

The objectives of this trial were: (1) to assess whether a mobile phone–enabled app could provide HIV patients with laboratory test results, (2) to better understand the implementation of such an intervention, and (3) to determine app effectiveness in improving linkage to HIV care after diagnosis.

**Methods:**

We developed and tested an app through a randomized controlled trial carried out in several primary health care facilities in Johannesburg. Newly diagnosed HIV-positive patients were screened, recruited, and randomized into the trial as they were giving a blood sample for initial CD4 staging. Trial eligibility included ownership of a phone compatible with the app and access to the internet. Trial participants were followed for a minimum of eight months to determine linkage to HIV care indicated by an HIV-related laboratory test result.

**Results:**

The trial outcome results are being prepared for publication, but here we describe the significant operational and technological lessons provided by the implementation. Android was identified as the most suitable operating system for the app, due to Android functionality and communication characteristics. Android also had the most significant market share of all smartphone operating systems in South Africa. The app was successfully developed with laboratory results sent to personal smartphones. However, given the trial requirements and the app itself, only 10% of screened HIV patients successfully enrolled. We report on issues such as patient eligibility, app testing in a dynamic phone market, software installation and compatibility, safe identification of patients, linkage of laboratory results to patients lacking unique identifiers, and present lessons and potential solutions.

**Conclusions:**

The implementation challenges and lessons of this trial may assist future similar mHealth interventions to avoid some of the pitfalls. Ensuring sufficient expertise and understanding of the programmatic needs by the software developer, as well as in the implementation team, with adequate and rapid piloting within the target groups, could have led to better trial recruitment. However, the majority of screened patients were interested in the study, and the app was installed successfully in patients with suitable smartphones, suggesting that this may be a way to engage patients with their health care data in future.

**Trial Registration:**

ClinicalTrials.gov NCT02756949; https://clinicaltrials.gov/ct2/show/NCT02756949 (Archived by WebCite at http://www.webcitation.org/6z1GTJCNW)

## Introduction

Joint United Nations Programme on HIV/AIDS (UNAIDS) and World Health Organization have driven the “90-90-90” initiative since 2015, to maximize the impact of expanded antiretroviral therapy (ART) coverage for both individual health and for decreasing new infections among the population. The initiative calls for 90% of HIV positive people to know their status, 90% of those who are eligible for ART to be initiated on ART, and 90% of those on ART to maintain viral suppression [1].

The *HIV care cascade* shows significant patient attrition between HIV diagnosis and entry into HIV care [2]. This means delayed access to ART, with avoidable illness and mortality, as well as unnecessary transmission of the virus. Interventions have been tested to decrease this attrition, most involving expediting HIV or CD4 (cluster of differentiation) T-cell staging results by health workers [3]. In a 2015 cohort study in South African primary health care facilities, only 64% of persons newly diagnosed with HIV who had been assessed for CD4 staging returned to the clinic for the CD4 result [4]. Improved access to the point of care CD4 counts, widely anticipated to increase linkage to HIV care, has yielded limited success, suggesting that other types of interventions are required, [5] including interventions for patient empowerment and better patient involvement in their own care.

Interest in using cell phones to facilitate health care has increased in recent years in Africa, due to high levels of cell phone ownership [6]. Recent evidence using short message service (SMS) text messaging showed modest adherence benefits [7,8,9]. Mobile apps on “smart” mobile phones, have become a major part of people’s lives, from providing simple access to information (eg, Google), Global Positioning System (GPS) tracking and engagement with services (eg, Uber), to banking (eg, all South African banks), social media (eg, Facebook), and instant messaging (eg, WhatsApp). We are unaware of any study that has used a mobile phone app to link HIV positive patients to care.

We conducted a randomized controlled trial in multiple Johannesburg HIV testing sites, recruiting between October 2015 and June 2016 and following up till February 2017, to test whether providing newly diagnosed HIV patients their laboratory results and supporting information securely on their mobile phones, via an app, would improve linkage to HIV care [10]. This article reflects on the significant operational challenges in the project, including data linkage, software design, trial eligibility, interoperability of systems and devices, and general management and delivery of the intervention. The results on whether the intervention was effective in improving linkage to care will be published as soon as they become available.

## Methods

### Setting

South Africa has the world’s largest number of people living with HIV, with antiretroviral care provided freely through the state sector to over 3.5 million people [11]. The country has formal HIV testing, staging (CD4) and care guidelines, followed by all public health facilities, with a National Health Laboratory System (NHLS) providing laboratory testing for the majority of South Africans, including for the HIV program [12,13]. HIV testing is largely facility-based and conducted by lay counselors, with blood drawn for a CD4 count test after a positive HIV diagnosis is made and the patient told to return to a health facility. Around 350,000 people annually are started on ART in South Africa [11].

The trial was conducted by Wits Reproductive Health and HIV Institute, an academic research and program implementation organization, in partnership with the NHLS and the World Bank, in public sector clinics and hospitals in inner-city Johannesburg, a densely populated urban area with a well-established HIV testing and ART program. This inner-city location is an area with significant levels of immigration, unemployment, alcohol abuse, sex work, poverty, and gender violence. We were specifically interested in sampling young people between the age of 18 and 30 years and men, representing two key groups under-represented in South African treatment program, and with poorer linkage to care [1,11].

### Mobile Phone Variety, App Design, and Data Linkage

A preliminary assessment conducted from March-April 2015 at the largest recruitment site found 160/373 (42.9%) of patients had mobile phones, which matches the reported national data statistics of 35%-50% [13]. Apps have to be designed for different operating systems or “platforms” (eg, Android, iOS). It’s an evolving area as phone manufacturers upgrade and move between platforms. The locally popular Blackberry and Nokia phones have recently switched to an Android platform and carry different skills regarding development. The most common mobile phone operating system from our formative research phase of the trial was Android 67/158 (42.4%) followed by Blackberry OS, Windows Phone, WebOS, Firefox OS and Symbian, each with less than 20%. The iPhone ownership was extremely low at 4/158 (2.5%). Similar results were shown in a survey done by the project team in early 2016 in the Eastern Cape, a far more rural province, where an even greater proportion of patients had Android devices, probably as Blackberry and Nokia by then had stopped making less expensive mobile phones for the local market.

The decision to use the Android platform for the app development was based on several issues beyond Android-based phones were the most common. Android functionality allowed for third-party “push” notifications, had secure app-based data transmission essential for laboratory results, both of which were a key aspect of the intervention. Additionally, resource constraints did not allow for development of additional platforms, most of which had been declining in popularity for years.

Developing the app, which was called SmartLink, consisted of selecting the necessary features for the study, creating the app layout and the health content, combining the features and the health content in the app layout, and conducting field testing. The app developer was selected by an existing relationship and was identified to be suitable for the task based on experience with a similar project involving interfacing an Android app with NHLS laboratory data. The developer was provided with a user interface outline developed by the research team.

**Figure 1:**
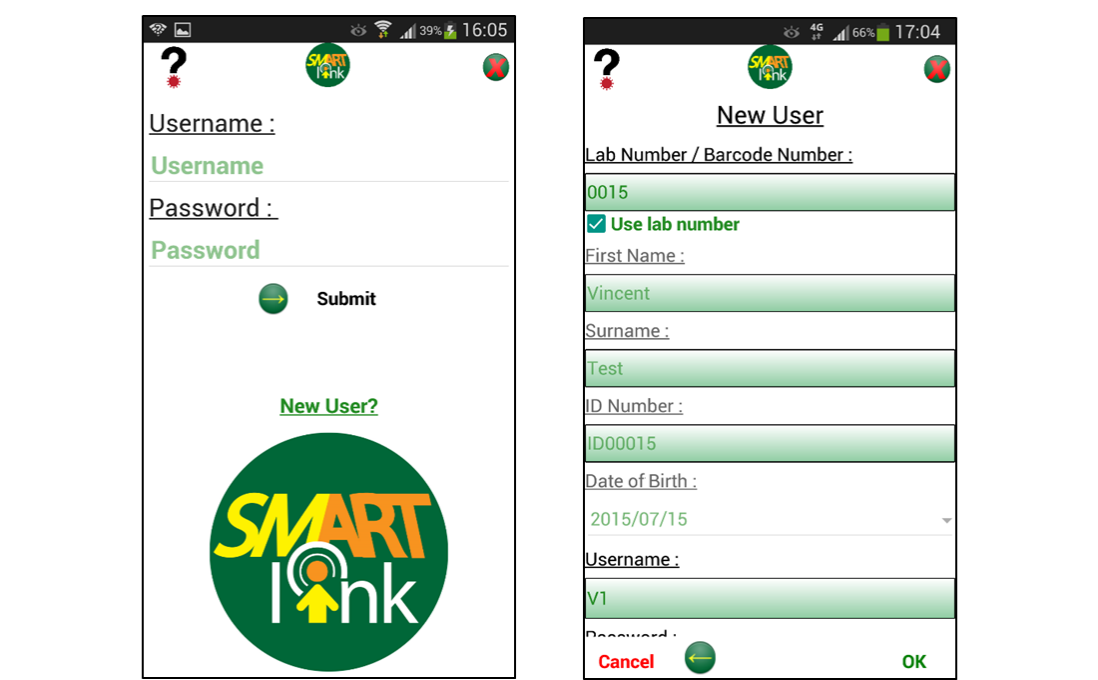
Screenshots of the SmartLink app, showing the login procedure and illustrative home screen.

**Figure 2:**
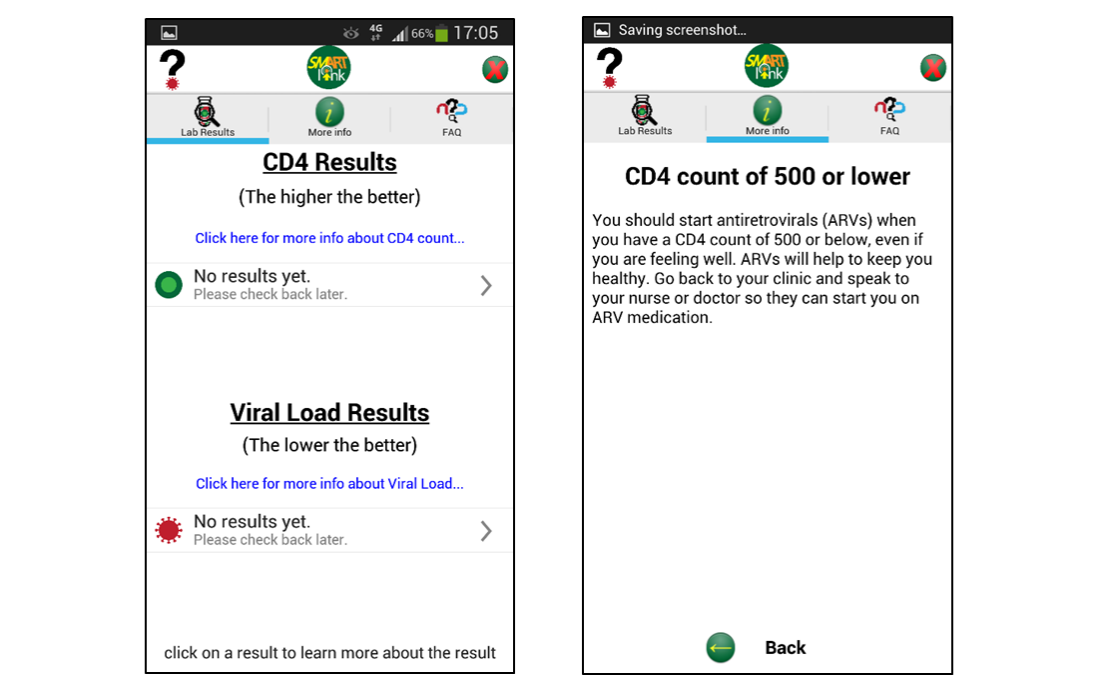
Screenshots of the SmartLink app, illustrating the results format and an example of advice given.

App content was created by HIV clinicians and an experienced HIV psychosocial support team, who also included graphics to better convey information. The app was developed to be as compact as possible, while also being interactive, dynamic and informative for patients. Laboratory results were color-coded, based on red-yellow-green traffic lights, to help patients quickly understand their results. Images were used to split up larger pieces of informational text, while also providing a visual example of relevant topics (Figures 1 and 2). Development of the app took approximately two and a half months including field testing. Individuals within the study team were used to test installation of the app on Android cell phones. Members of a community advisory group including some living with HIV who use public health facilities tested the app for usability.

Although field testing was done, the final app was not placed on Google Play Store, an access point for downloadable apps for Android devices, due to possible disclosure issues. The app would only be downloaded by confirmed HIV positive people; this meant any software updates needed to be done manually on individual phones, rather than through the Google Play app store. SmartLink had a final installation size of 12.5 MB and intended to work on any version of Android version 4.2 or higher and on any phone with at least 350 MB of random access memory (RAM).

Free internet is available in selected public urban areas within South Africa, but this varies widely regarding geography and reliability and is often limited in speed and time. The vast majority of internet users use their mobile phone with a prepaid system for data usage through their mobile network provider and are acutely aware of the costs of usage. Within the app, all reference material, images, and contact information are built in the initial download. For the study, SmartLink was installed by study staff from an Android install file and Wi-Fi dongle which allowed access to the installation file at no cost to the participant. This meant that app users only needed to download laboratory results, resulting in minimal data usage, which was key for app acceptability. Once installed, it was calculated that throughout the year of the study, data transfer would cost the participant less than 1 Rand (<US $0.10). Participants received a 50 Rand (≈US $4) phone credit at recruitment to ensure all app-related costs were covered.

### Intervention

Newly diagnosed HIV-positive patients were recruited to the study by trained study staff at five local HIV testing sites, in and around inner-city Johannesburg. The testing sites consisted of three public health clinics, a provincial community health center, and later in the study a tertiary hospital. Inclusion criteria were: being newly diagnosed with HIV, being a local resident, aged 18 years and older, not pregnant on the date of recruitment, able to read English or isiZulu (ie, the most commonly used local language), carrying a photo identification (ID) during prescreening, and having a data-enabled mobile phone with Android 4.2 or higher and sufficient RAM to allow app installation, done during screening). Staff responsible for recruitment were provided with training on the study protocols and the use of Research Electronic Data Capture, a data collection and participant randomization system, on a tablet device.

Eligible and consenting participants were randomized to either the app intervention arm or the standard-of-care control arm of the trial, where participants were simply referred to their local ART initiation site where they could collect their CD4 result and initiate treatment if needed. Simple socio-economic and phone ownership data were collected from all newly diagnosed HIV patients during eligibility screening. Our statistical sample size calculation suggested 1000 participants in each arm would give us sufficient power to evaluate the rates of linkage to HIV care the two subgroups (ie, men and younger participants).

Project staff assisted participants randomized to the app arm with the installation of SmartLink and shown how to view their laboratory results and access HIV related support information. At the time of enrolment, app use was relatively low in this population, and may not be necessary today. The app was accessible using a password and personal information number (PIN) system, as used by the local banks, preventing access to confidential information by third parties with access to the phone; initial access to the app required the user to insert both the password and the PIN while subsequent access just required the PIN. The app was set up to communicate two laboratory results, CD4 count and viral load, in simple language which included the date and time, a visual colored-coded scale and “normal” values, and a short explanation of what the result means and what action, if any, should be taken. The app also provided participants with access to information about HIV, ART adherence, and laboratory tests in English and isiZulu.

At the time of the study (2015-2016), the South African CD4 ART initiation threshold was 500 cells/µl; after a positive HIV test, patients had a blood draw for CD4 staging and were told to return for the result, and ART started based on the CD4 count (<500 cells/µl), clinical status, and treatment readiness. As part of informed consent and regardless of study arm, all participants were instructed to attend their clinics within the next few weeks for a follow-up, as per the instructions given to them by the clinic staff, and not simply wait for results on their phone for the intervention arm.

The trial protocol obtained approval from the University of Witwatersrand’s Medical Human Research Ethics Committee, the City of Johannesburg and Gauteng’s Department of Health at the provincial level and was registered in ClinicalTrials.gov (NCT02756949). All participants provided written informed consent before enrolment.

## Results

The study screened over 4500 patients at five HIV testing sites, but only randomized 353/4537 (7.78%) participants. There were some diverse challenges in implementing the study, which significantly impacted on the speed with which the study was implemented, as well as the number of participants recruited.

### Recruitment and Eligibility

In 2015, before the trial commenced, between 40 and 90 new HIV cases were being identified daily [14]. Based on this, the study team estimated that three months of study recruitment would be sufficient to meet the study sample requirement. For example, 17 individuals recruited per day for three months for a total of 1000 participants across all study sites.

However, when we began the study in October 2015, the number of new HIV cases had dropped to 10-15 per day, and recruitment was therefore compromised. This is likely due to the introduction of decentralized testing to numerous surrounding facilities at the time of the trial. To increase recruitment, the protocol was changed to add a nearby busy HIV testing site at the tertiary hospital, but the local site negotiation and reapplication to regulators meant this site came on very late, contributing only 24.9% (88/353) of the participants, and thus could not compensate for the low absolute recruitment number.

During study recruitment, from October 12, 2015, to June 17, 2016, 4537 individuals were identified as being HIV positive (Table 1). Of those, 3540/4537 (78.02%) were willing to participate in the trial, were aged over 18 years, could read English or isiZulu, had a photo ID to confirm their identity, and had access to a mobile phone, with 754/3540 (21.30%) having an Android mobile phone with data access. Approximately 1.92% (87/4537) of people could not participate as they did not read English or IsiZulu.

**Table 1:** Recruitment cascade with reasons for ineligibility.

Characteristics		Participants, n (%)^a^
**Prescreened (HIV-positive)**		4537 (100.0)
	Declined participation	90 (2.0)
	Under 18 years old	12 (0.3)
	Pregnant	269 (5.9)
	Cannot read English or isiZulu	87 (1.9)
	No photo identification (before requirement removed on 17 December 2015)	539 (11.9)
Passed prescreening		3540 (78.0)
**Screened**		3540 (100.0)
	No working phone	498 (14.1)
	No active SIM^b^ card	8 (0.3)
	No android phone	2100 (59.3)
	Do not use data	226 (6.4)
	Insufficient RAM^c^	133 (3.8)
	Android version lower than 4.2	222 (6.3)
Passed screening		353(10.0)
**Randomized**		353 (100.0)
	Intervention (mobile app)	181 (51.3)
	Standard of care (control)	172 (48.7)

^a^Totals might not add to 100% due to decimal rounding.

^b^SIM: subscriber identification module.

^c^RAM: random access memory.

**Table 2:** Reasons for initial refusal to enrol in study (N=90).

Reason	n (%)
Not interested	30 (33.7)
Not ready to discuss/disclose status	18 (20.2)
In a hurry	16 (18.0)
Sick and not able to talk	6 (6.7)
Do not feel comfortable	5 (5.6)
In denial	3 (3.4)
Other	9 (9.0)
Blank/missing reason	3 (3.4)

**Figure 3:**
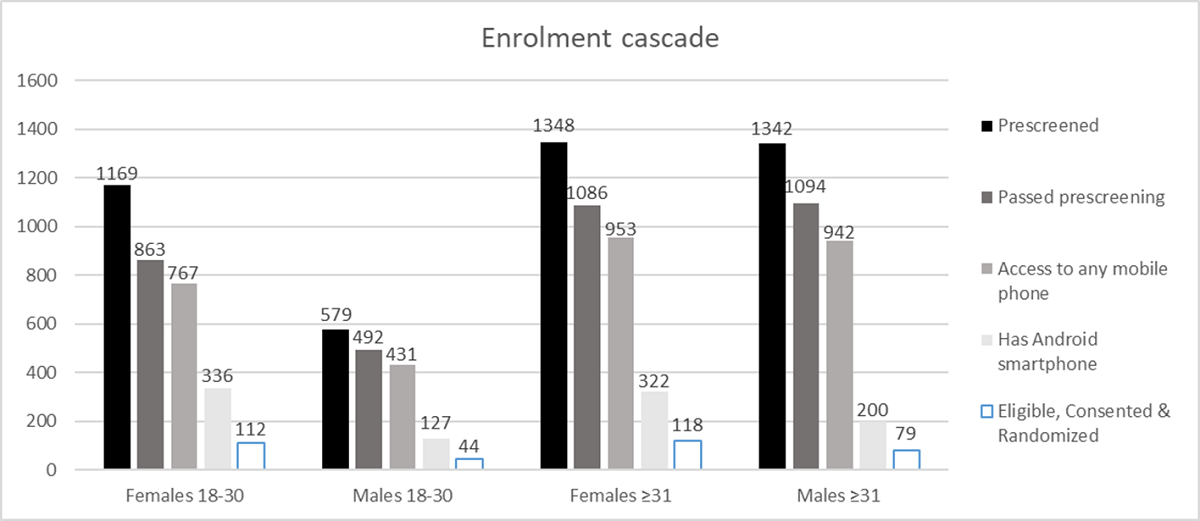
Enrolment cascade (number of participants).

A proportion of trial candidates did not have a working phone or active subscriber identification module (SIM) card (506/3540, 14.29%). Not having an Android mobile phone was the single most important factor of ineligibility for the trial (2100/3540, 59.32%), and this was compounded by related factors such as not having the correct Android version, adequate RAM or mobile data (581/3540, 16.41%). A small number were not willing to participate in the study, for the reasons listed in Table 2. The enrolment cascade by age group and gender is shown in Figure 3. Only 44/3540 (1.24%) young men were recruited, a key target group for the study.

### Photo Identification

Enrolment in the study initially required verification of identity using photo ID due to the concern that without photo ID it would be difficult to verify the true identity of a person and therefore there would be a risk of disclosing confidential laboratory information to the wrong person. However, almost two thirds (529/824, 64.2%) of the patients screened did not have a photo ID at trial screening. The requirement for photo ID was therefore removed from the study eligibility criteria, with the consent of the Human Research Ethics Committee on 17 December 2015. To address the aforementioned disclosure concern the study team manually checked the NHLS database for each participant at the time of enrolment to ensure that the participant was new to the NHLS system and therefore would not have access to confidential laboratory results of a potentially different person. Access to the NHLS database was limited to preauthorised study personnel.

### App Design and Mobile Phone Platforms

The projected percentage of Android phone ownership was as anticipated through the life of the trial. However, the features required by the final version of the app, including security settings, limiting access, and live notifications sent to users, required a relatively new version of Android that was not supported by more than a third (222/575, 38.6%) of the Android phones our participants used, hampering eligibility for trial recruitment. The research project team met or spoke with the developer on an almost weekly basis during the design, implementation and analysis phase of the project. Many of the concerns regarding the app only became apparent as the study was being implemented, and capacity to rectify these were limited by the project timelines. Some mobile phones, despite being modern and supporting the up-to-date Android platform, did not have adequate RAM to allow app installation (Table 2) and we, therefore, had to exclude an additional 133/3540 (3.76 %) patients as a result. RAM is an expensive part of the phone, and cheaper smartphones had smaller levels of RAM, a problem we did not anticipate. The next unexpected challenge was that the app, despite field testing from the developer on one type of tablet, did not work on Android tablet devices that some patients had, again limiting recruitment, although this only affected a small number of trial candidates (n=15).

These problems were identified after the study had started. At that time recruitment was already severely behind schedule and the study team therefore elected to proceed with the study. As previously mentioned, the app was not made available through portals such as Google Play Store, as the study was HIV-specific and hence a download would have violated confidentiality around diagnosis. The Play Store has compatibility checking algorithms that are used before products are allowed to be released through the platform; this would likely have made explicit the app’s minimum requirements earlier and reduced the number of issues we encountered. Without this check, the study team had to make the developer aware each time a problem was identified after the study was initiated.

### Data Systems and Interoperability

A unique cross-facility patient identifier not available in the clinic or NHLS’s patient information systems. This meant that the investigators had to create a method to keep track of trial participants and their laboratory results across multiple databases that did not communicate directly with each other. As a result, any single error in patient information collection and transcription during recruitment, clinic blood specimen collection or blood analysis led to difficulties in identifying that patient in the other databases. Throughout the trial, data quality processes were challenging because of the manual process of searching for participant information in NHLS’s TrakCare user interface. The investigators found that 20.4% (72/353) of participants who had initially been identified as lost to follow up (ie, no follow up CD4 or viral load) using the laboratory request data which includes names and birth dates were actually linked to care at 8 months post-recruitment when a manual search of the NHLS database was conducted that included variations of names and dates of births. The different NHLS and research trial REDCap data systems all relied on positive ID of individual patients but lacked a unique patient ID between the systems.

Full interoperability for data exchange between the relevant systems and devices could not be achieved during the trial. A substantial number of participants in the trial arm did not receive their laboratory data through SmartLink, despite the successful installation of the app, and despite the data being available within the NHLS database. We are still working with the software designer to try to understand the causes of this problem. While participants were verbally instructed during trial enrolment to attend their clinics according to the standard South African HIV care and treatment protocol that all newly diagnosed patients receive and not simply wait for results on their app, the fact that some results were not received via the app does compromise the trial assessment of the effectiveness of the app.

## Discussion

### Principal Findings

A mobile phone–based intervention in urban South Africa still excludes at least 40% of newly diagnosed persons living with HIV simply based on the fact that they do not own or have access to a mobile phone. This combined with other trial entry requirements, such as needing to have Android 4.2 version or higher, app installation space and data, and the lower number of new HIV cases due to decreasing yield and decentralization of HIV testing made meeting the sample size requirement a challenge. In the end, less than 10% of people newly diagnosed with HIV at the study sites who were screened for the trial were enrolled in the study, although interest in participating in the study was high, with only 90/4537 (1.98%) people approached not wanting to join. Over 4500 new HIV cases were screened for trial participation, but only 353/4537 (7.78%) could be randomized. Of concern, the app only worked on more expensive or newer mobile phones, disadvantaging poorer patients.

Creating apps is complex and requires attention to an evolving technological environment. The software developer selected had a prior engagement with the NHLS and came recommended. Despite the fact that a number of the project team members were experienced in working in mobile health (mHealth), only one person, working within the NHLS, had experience in app creation—a tuberculosis (TB) app used by nurses and specifically designed for tablets, allowing for dynamic notifications, scheduling future notifications and matching patient data from two databases without a unique identifier. Additionally, the software development company that was hired to create our app had much experience with databases but very limited experience creating Android apps.

The team’s inexperience with working with studies centralized around Android app–based interventions, as well as the difficulty in the relationship with the developer, meant that there was little ability to manoeuvre within the tight project timelines. This lack of experience from the project and Android software development teams likely contributed to the multiple app-creation challenges encountered. Having independent mechanisms to assess software development and technical support to the process by more expert teams would have been helpful.

The implications of these challenges are that any future similar app design or upgrade has to take the different platforms and phone operating systems into account, based on shifting preference for different phones. At the moment, Android apps have the maximum market share, and this was the platform we elected to use. Additional advantages of Android systems are that they allow for third-party push communication, their security systems ensure secure transmission and viewing of personal health data, and programming is easier compared to other operating systems. SmartLink itself has the potential to meet all these requirements if done by an experienced Android app developer.

**Table 3:** Study challenges and solutions.

Challenges	Potential solutions
Phone compatibility, data availability especially for poorer participants and feasibility of implementation across databases	Adequate compatibility testing Enable innovative access to data (either access to free WIFI hotspots, or vouchers) Pilot implementation adequately with the target group
Lack of app availability across platforms	Resources available for development across common platforms, if platforms differ at the time of implementation
Poor app development and testing	Ensure sufficient expertise in app development and testing within the study team Implement “agile” software development approaches, including field testing on ‘’entry-level’’ commonly used mobile phone Use of access points (eg, Google Play Store) to quality check app
Manual installation of the app, with training	Use of access points for easier downloads Improve usability to minimize instruction
Recruitment speed	Ensure majority of the target population are eligible, through ensuring entry restrictions are minimized (data access, phone type, etc) Piloting should be done mainly on a target group, not proxies such as staff, advisory groups or participants within existing clinics
Lack of photo identification of potential study participants	Have alternative ways of registering patients against the database Utilise future single patient identifiers that allow for cross-database identification

Given its functionality, SmartLink’s graphics and code could be compressed to get the install size down to under 5 MB. This is a reasonable size of an app with the functions of SmartLink, and the code could be optimized to lower the minimum RAM requirements. Developers need to ensure that software used in lower resourced settings can be used on older and less powerful phones.

There was a lack of availability of photo ID. In future, if a similar intervention is being rolled out, it is probable that some verifiable ID will be required for people to have routine access to their laboratory results. Educating health care clients that they need to bring this form of ID with them will be required at scale. Ideally, a unique clinic ID that links to both the NHLS database and the app would be available.

In line with the Department of Health’s mHealth Strategy and National Adherence Guidelines, which advocates for the use of cell phone technology to improve patient experiences with health systems and adherence to medication and visit schedules, we had planned to expand the use of the app beyond HIV testing and linkage to care; initial expansion of the app was planned to ART clinics, where patients are stable on ART, to communicate routine monitoring results; in future, it was to be used to communicate follow up actions and medication availability [15,16]. There were also plans to extend the service to TB and diabetic patients and increase the app’s functionality to allow doctors and nurses at a clinic level to send messages to individual patients if laboratory results are unexpected. Finally, we planned to use the app to support research participants, allowing communication between the site and the participant, possibly allowing us to assess self-testing devices used by patients to monitor their own chronic condition. While all these aspirations still stand, the current app will require substantial upgrading and operational testing before being deployed in such a variety of situations.

A large number of operational issues encountered suggest that far more care at a project management level and sufficient software expertise are required before an expansion of this approach to improving linkage to care (Table 3). The research team is looking at new approaches that would be accessible to a greater proportion of the population.

### Conclusion

This study was trying to demonstrate something fundamental—that empowering patients with information would lead to more engagement in care, not that a specific technology works. The fact that the technology and operational issues were significant obstacles means we were not able to test our starting hypothesis.

Formal conventional scientific studies that evaluate mHealth interventions aiming to improve health outcomes suffer from the fact that the design and execution of these studies often take years, in an environment where changes in technology and social media are often measured in months. This project was far more complex than we anticipated, with multiple unanticipated challenges. Only a small minority of patients were able to access our intervention; those without mobile phones were excluded, and alternative methods may be required for these patients.

The importance of understanding health clients’ needs and preferences to design systems from a client perspective cannot be overemphasized. Conducting market research among clients with less access to health services and lower adherence should be prioritized. Learning from commercial sector systems especially mobile banking, which satisfy compliance standards and are widely used across socio-economic strata, may also be beneficial, as well as understanding the user experiences with such systems. Gradually building a module based mHealth system might work best, commencing with a component like client access to laboratory results, as in this trial, and expanding to the challenging and interlinked areas of laboratory results-dependent rescripting and drug refill schemes with participating pharmacies and clinics. If functional, these could relieve pressure from clinics and make long-term treatment more convenient to patients.

Mobile health approaches are exciting, with the potential to engage patients in their own care in a way that’s unprecedented. However, there is substantial work to be done on how to do this most effectively, and expertise in multiple areas of implementation is required.

## Appendix

Multimedia Appendix 1 CONSORT‐EHEALTH checklist (V 1.6.1).

## Abbreviations

ART: antiretroviral therapy
CD: cluster of differentiation
GPS: Global Positioning System
ID: identification
mHealth: mobile health
NHLS: National Health Laboratory System
PIN: personal information number
RAM: random access memory
SIM: subscriber identity module
SMS: short messaging services
TB: tuberculosis
UNAIDS: Joint United Nations Programme on HIV/AIDS
