# Supplementary material for: Improving Linkage to HIV Care Through Mobile Phone Apps: Randomized Controlled Trial
Source: JMIR Mhealth Uhealth. 2018 Jul 17;6(7):e155. doi: 10.2196/mhealth.8376 (PMC6068383; doi:10.2196/mhealth.8376)
Supplement: Multimedia Appendix 1 [file mhealth_v6i7e155_app1.pdf]

CONSORT-EHEALTH Checklist V1.6.2 Report	Manuscript Number	8376
**(based on CONSORT-EHEALTH V1.6), available at [http://tinyurl.com/consort-ehealth-v1-6].**		
**Date completed**		
7/9/2018 12:27:36		
**by**		
Willem Venter		
Improving Linkage to HIV Care Through Mobile Phone Apps: A Randomized Clinical Trial		
TITLE		
**1a-i) Identify the mode of delivery in the title**		
"" a Randomized Clinical Trial""		
**1a-ii) Non-web-based components or important co-interventions in title**		
**1a-iii) Primary condition or target group in the title**		
""HIV patients""		
ABSTRACT		
**1b-i) Key features/functionalities/components of the intervention and comparator in the METHODS section of the ABSTRACT**		
Linkage to care		
**1b-ii) Level of human involvement in the METHODS section of the ABSTRACT**		
Not covered in abstract		
**1b-iii) Open vs. closed, web-based (self-assessment) vs. face-to-face assessments in the METHODS section of the ABSTRACT**		
"Newly diagnosed HIV-positive patients were screened, recruited, and randomized into the trial as theywere giving a blood sample for initial CD4 staging. "		
**1b-iv) RESULTS section in abstract must contain use data**		
"only 10% of screened HIV patients successfully enrolled""		
**1b-v) CONCLUSIONS/DISCUSSION in abstract for negative trials**		
"The implementation challenges and lessons of this trial may assist future similar mHealth interventions to avoidsome of the pitfalls"		
INTRODUCTION		
**2a-i) Problem and the type of system/solution**		
"..test whether providing newly diagnosed HIV patients theirlaboratory results and supporting information securely on theirmobile phones, via an app, would improve linkage to HIV care""		
**2a-ii) Scientific background, rationale: What is known about the (type of) system**		
""We are unaware of any studythat has used a mobile phone app to link HIV positive patients to care"		
**Does your paper address CONSORT subitem 2b?**		
This article reflects on the significant operationalchallenges in the project, including data linkage, softwaredesign, trial eligibility, interoperability of systems and devices,and general management and delivery of the intervention"		
METHODS		
**3a) CONSORT: Description of trial design (such as parallel, factorial) including allocation ratio**		
""We conducted a randomized controlled trial in multipleJohannesburg HIV testing sites, recruiting between October2015 and June 2016 and following up till February 2017, to test whether providingnewly diagnosed HIV patients their laboratory results and supporting information securely on theirmobile phones,<br>via an app, would improve linkage to HIV care""		

**3b) CONSORT: Important changes to methods after trial commencement (such as eligibility criteria), with reasons**		
We expanded the catchment area to additional facilities, as recruitment was too slow in the idientifed clinics		
**3b-i) Bug fixes, Downtimes, Content Changes**		
No		
**4a) CONSORT: Eligibility criteria for participants**		
Newly diagnosed HIV-positive patients over 18 years		
**4a-i) Computer / Internet literacy**		
Had to be able to use a smart phone app		
**4a-ii) Open vs. closed, web-based vs. face-to-face assessments:**		
"Newly diagnosed HIV-positive patients were recruited to the study by trained study staff at five local HIV testing sites, in and around inner-city Johannesburg"		
**4a-iii) Information giving during recruitment**		
Inform consent procedures wre as epr legal regulatory requirements		
**4b) CONSORT: Settings and locations where the data were collected**		
Johannesburg		
**4b-i) Report if outcomes were (self-)assessed through online questionnaires**		
""Inner city Johannesburg""		
**4b-ii) Report how institutional affiliations are displayed**		
Not addressed		
**5) CONSORT: Describe the interventions for each group with sufficient details to allow replication, including how and when they were actually administered**		
**5-i) Mention names, credential, affiliations of the developers, sponsors, and owners**		
not addressed or detailed		
**5-ii) Describe the history/development process**		
"Developing the app, which was called SmartLink, consisted of<br>selecting the necessary features for the study, creating the applayout and the health content, combining the features and thehealth content in the app layout, and conducting field testing""		
**5-iii) Revisions and updating**		
Not addressed		
**5-iv) Quality assurance methods**		
Not addressed		
**5-v) Ensure replicability by publishing the source code, and/or providing screenshots/screen-capture video, and/or providing flowcharts of the algorithms used**		
Screenshots are in the manuscript		
**5-vi) Digital preservation**		
Not addressed		
**5-vii) Access**		
" For the study, SmartLink was installed bystudy staff from an Android<br>install file and Wi-Fi dongle which allowed access the installation file at no cost to the participant"		
**5-viii) Mode of delivery, features/functionalities/components of the intervention and comparator, and the theoretical framework**		
This is only covered very superficially		
**5-ix) Describe use parameters**		
No		

**5-x) Clarify the level of human involvement**		
Counslleors assisted with app installation		
**5-xi) Report any prompts/reminders used**		
SMS prompts		
**5-xii) Describe any co-interventions (incl. training/support)**		
None		
**6a) CONSORT: Completely defined pre-specified primary and secondary outcome measures, including how and when they were assessed**		
We did not clarify these in any detail, as this was not the primary aim of this paper; linkage to care was the primary outcome, though, and usability and operational lessons were secondary objectives		
**6a-i) Online questionnaires: describe if they were validated for online use and apply CHERRIES items to describe how the questionnaires were designed/deployed**		
No		
**6a-ii) Describe whether and how "use" (including intensity of use/dosage) was defined/measured/monitored**		
Numbers of times the app was opened		
**6a-iii) Describe whether, how, and when qualitative feedback from participants was obtained**		
Not assessed		
**6b) CONSORT: Any changes to trial outcomes after the trial commenced, with reasons**		
Johannesburg		
**7a) CONSORT: How sample size was determined**		
**7a-i) Describe whether and how expected attrition was taken into account when calculating the sample size**		
""Our statistical samplesize calculation suggested 1000 participants in each arm would give us sufficient power to evaluate the rates of linkage to HIV care the two subgroups (ie, men and younger participants)."		
**7b) CONSORT: When applicable, explanation of any interim analyses and stopping guidelines**		
We did not clarify these in any detail, as this was not the primary aim of this paper; linkage to care was the primary outcome, though, and usability and operational lessons were secondary objectives		
**8a) CONSORT: Method used to generate the random allocation sequence**		
Not applicable		
**8b) CONSORT: Type of randomisation; details of any restriction (such as blocking and block size)**		
Individual randomization		
**9) CONSORT: Mechanism used to implement the random allocation sequence (such as sequentially numbered containers), describing any steps taken to conceal the sequence until interventions were assigned**		
Not detailed here; was done with simple random number allocation		
**10) CONSORT: Who generated the random allocation sequence, who enrolled participants, and who assigned participants to interventions**		
Study staff generated the sequence, and did the assigning		
**11a) CONSORT: Blinding - If done, who was blinded after assignment to interventions (for example, participants, care providers, those assessing outcomes) and how**		
**11a-i) Specify who was blinded, and who wasn't**		
No one was blinded		
**11a-ii) Discuss e.g., whether participants knew which intervention was the "intervention of interest" and which one was the "comparator"**		
Was covered in consent form		
**11b) CONSORT: If relevant, description of the similarity of interventions**		
Comparritor was the standard of care - simple referral		
**12a) CONSORT: Statistical methods used to compare groups for primary and secondary outcomes**		

Not applicable fro this paper		
**12a-i) Imputation techniques to deal with attrition / missing values**		
Not applicable fro this paper		
**12b) CONSORT: Methods for additional analyses, such as subgroup analyses and adjusted analyses**		
Not applicable fro this paper		
RESULTS		
**13a) CONSORT: For each group, the numbers of participants who were randomly assigned, received intended treatment, and were analysed for the primary outcome**		
353 randomised, final analysis for future manuscript		
**13b) CONSORT: For each group, losses and exclusions after randomisation, together with reasons**		
Not relevant to this manuscript		
**13b-i) Attrition diagram**		
Not relevant to this manuscript		
**14a) CONSORT: Dates defining the periods of recruitment and follow-up**		
"study recruitment, from October 12, 2015, to June 17,2016"; length of followup not relevant to this manuscript		
**14a-i) Indicate if critical "secular events" fell into the study period**		
Not applicable		
**14b) CONSORT: Why the trial ended or was stopped (early)**		
Study ended as funding at an end		
**15) CONSORT: A table showing baseline demographic and clinical characteristics for each group**		
In a table in manuscript		
**15-i) Report demographics associated with digital divide issues**		
Adressed in a table		
**16a) CONSORT: For each group, number of participants (denominator) included in each analysis and whether the analysis was by original assigned groups**		
**16-i) Report multiple "denominators" and provide definitions**		
We under-recruited in the interest groups, and generally, so were not able to analyse these		
**16-ii) Primary analysis should be intent-to-treat**		
Not relevant to this manuscript		
**17a) CONSORT: For each primary and secondary outcome, results for each group, and the estimated effect size and its precision (such as 95% confidence interval)**		
Not applicable to this manuscript		
**17a-i) Presentation of process outcomes such as metrics of use and intensity of use**		
Not applicable to this manuscript		
**17b) CONSORT: For binary outcomes, presentation of both absolute and relative effect sizes is recommended**		
Not applicable to this manuscript		
**18) CONSORT: Results of any other analyses performed, including subgroup analyses and adjusted analyses, distinguishing pre-specified from exploratory**		
Not applicable to this manuscript		
**18-i) Subgroup analysis of comparing only users**		
Not applicable to this manuscript		
**19) CONSORT: All important harms or unintended effects in each group**		
Not applicable to this manuscript		

**19-i) Include privacy breaches, technical problems**		
Addressed in detail throughout the manuscript		
**19-ii) Include qualitative feedback from participants or observations from staff/researchers**		
Not applicable to this manuscript		
DISCUSSION		
**20) CONSORT: Trial limitations, addressing sources of potential bias, imprecision, multiplicity of analyses**		
**20-i) Typical limitations in ehealth trials**		
Not blinded, and under-powered		
**21) CONSORT: Generalisability (external validity, applicability) of the trial findings**		
**21-i) Generalizability to other populations**		
Not applicable to this manuscript; generalizability will be somewhat limited - woendrship of a smart phone, able to have access to this form of app and data		
**21-ii) Discuss if there were elements in the RCT that would be different in a routine application setting**		
No, the design was there to be as real world as we could make it		
**22) CONSORT: Interpretation consistent with results, balancing benefits and harms, and considering other relevant evidence**		
**22-i) Restate study questions and summarize the answers suggested by the data, starting with primary outcomes and process outcomes (use)**		
"This project was far morecomplex than we anticipated, with multiple unanticipatedchallenges. Only a small minority of patients able to access our intervention; those without mobile phoneswere excluded,and alternative methods may be required for these patients"		
**22-ii) Highlight unanswered new questions, suggest future research**		
No, our paper simply suggests the original question remains relevant		
Other information		
**23) CONSORT: Registration number and name of trial registry**		
as above		
**24) CONSORT: Where the full trial protocol can be accessed, if available**		
Not available		
**25) CONSORT: Sources of funding and other support (such as supply of drugs), role of funders**		
World Bank, with staff that assisted with study design and manuscript preparation		
**X26-i) Comment on ethics committee approval**		
""The trial protocol obtained approval from the University of Witwatersrand’s Medical Human Research Ethics Committee,the City of Johannesburg and Gauteng’s Department of Healthat the provincial level and was registered in ClinicalTrials.gov(NCT02756949). All participants provided written informed consent before enrolment."		
**x26-ii) Outline informed consent procedures**		
""All participants provided written informed consent before enrolment:		
**X26-iii) Safety and security procedures**		
"Although field testing was done, the final app was not placed on Google Play Store, an access point for downloadable apps for Android devices, due to possible disclosure issues. "		
**X27-i) State the relation of the study team towards the system being evaluated**		
Not relevant to this manuscript		
